# PARP1 hyperactivation: mechanisms and cellular consequences

**DOI:** 10.1042/BST20260683

**Published:** 2026-11-12

**Authors:** Pedro Ortega, Rémi Buisson

**Affiliations:** 1 Department of Biological Chemistry, School of Medicine, University of California Irvine, Irvine, CA, U.S.A.; 2 Department of Pharmaceutical Sciences, School of Pharmacy & Pharmaceutical Sciences, University of California Irvine, Irvine, CA, U.S.A.

**Keywords:** Condensate, DNA damage response, DNA repair, PARP1, RNA export, RNA localization

## Abstract

Poly(ADP-ribose) polymerase 1 (PARP1) is a key DNA-damage sensor that promotes DNA repair by synthesizing poly(ADP-ribose) (PAR), which recruits repair factors, chromatin regulators, and other PAR-binding proteins to DNA lesions. Because PAR shares structural features with RNA, it can engage RNA-binding domains and compete with RNA for common binding surfaces, extending PARP1 signaling beyond DNA repair to regulate RNA-binding proteins (RBPs) and RNA biogenesis. Here, we discuss the mechanisms that drive PARP1 hyperactivation and highlight how excessive PAR synthesis disrupts RNA metabolism, biomolecular condensation, and protein synthesis. Although PARP1 responds to a broad range of DNA damage, the magnitude of its activation is determined by lesion identity rather than overall DNA damage levels. Mechanistically, distinct forms of genotoxic stress, including alkylation, oxidative stress, and ATR inhibition, converge on the generation of abasic sites that APE1 cleaves during base excision repair, producing DNA breaks with 5′-deoxyribose phosphate termini that hyperactivate PARP1. High PAR levels disrupt RBP–RNA interactions, impair RNA export and splicing, and promote aberrant nuclear condensate formation, depleting cytoplasmic mRNA and suppressing translation. In parallel, sustained PARP1 hyperactivation can drive NAD^+^ and ATP depletion and promote parthanatos, a PARP1-dependent form of regulated cell death implicated in neurodegenerative diseases.

## Structure, recognition, and degradation of poly(ADP-ribose)

Poly(ADP-ribose) polymerase 1 (PARP1) is the founding member of the PARP family and a major sensor of DNA damage [1]. PARP1 is activated by a broad spectrum of DNA lesions, including single-strand breaks (SSBs), double-strand breaks (DSBs), and bulky DNA adducts [2]. Upon binding DNA lesions via its zinc finger domains, PARP1 becomes catalytically active and transfers ADP-ribose units from NAD^+^ onto target proteins, including PARP1 itself, releasing nicotinamide as a byproduct [2–4]. PARP1-mediated ADP-ribosylation promotes the recruitment of DNA repair factors and local chromatin remodeling, which collectively orchestrate the DNA damage response, facilitating efficient DNA repair and thereby preserving genome integrity and cell survival [2]. However, aberrant or uncontrolled PARP1 activity converts this protective response into pathological hyperactivation. PARP1 hyperactivation drives profound changes in nuclear architecture, RNA metabolism, and cellular bioenergetics by consuming NAD^+^ and ATP [5]. Rather than promoting cell survival, PARP1 hyperactivation ultimately triggers cell death and is associated with neurodegeneration [6].

### Poly(ADP-ribose): a nucleic acid-like biopolymer

PARP1 catalyzes the transfer of either a single ADP-ribose unit, termed mono-ADP-ribosylation (MARylation, MAR), or multiple ADP-ribose units to generate poly(ADP-ribose) (PARylation, PAR) [7–9]. Structurally, PAR is similar to RNA because both polymers are built from repetitive units of phosphate groups, riboses, and adenine. Specifically, each PAR unit contains two phosphate groups and two riboses per adenine, whereas each RNA nucleotide contains one phosphate group and one ribose per adenine (Figure 1A) [3]. As a result, a PAR 3-mer is comparable in backbone length to an RNA 6-mer [10]. However, unlike RNA, which is an exclusively linear polymer, PAR chains can adopt either linear or branched architectures (Figure 1B). PAR is a highly multivalent polymer capable of simultaneously engaging numerous proteins, promoting the recruitment of hundreds of proteins to DNA damage sites involved in DNA repair, chromatin remodeling, and RNA metabolism [8,9,11,12]. Because PAR and RNA share key structural features, many RNA-binding proteins (RBPs) can interact with both polymers. Consequently, the high levels of PAR generated during PARP1 hyperactivation compete with RNA for binding.

**Figure 1:**
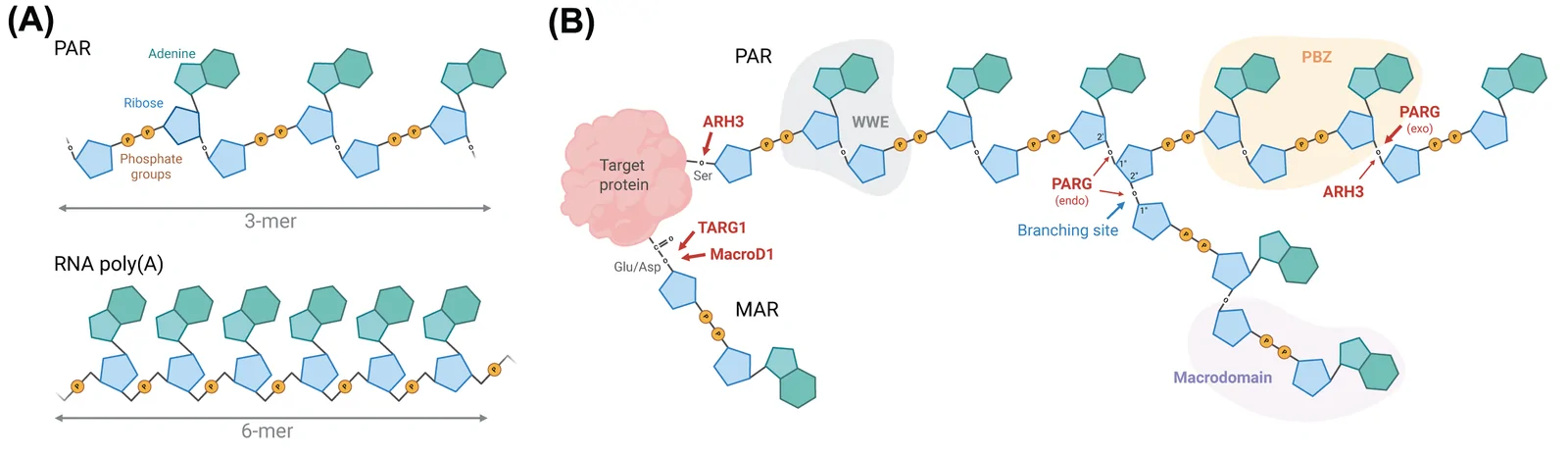
Structure of PAR and its recognition by PAR-binding modules (**A**) Schematic comparison of the chemical structures of PAR and poly(A) RNA. (**B**) The chemical structures of MAR and PAR conjugated to a target protein at the indicated amino acid residues are shown. Distinct PAR-binding modules recognize specific structural features of the polymer. The sites of action of the PAR erasers Poly(ADP-ribose) glycohydrolase (PARG), ARH3, TARG1, and MacroD1 are indicated, illustrating how they hydrolyze PARP1-mediated MAR or PAR at different positions to generate shorter PAR chains or free ADP-ribose.

Mechanistically, PAR synthesis by PARP1 proceeds through three chemically distinct reactions: initiation, elongation, and branching [13]. Initiation involves the transfer of the first ADP-ribose unit from NAD^+^ to an amino acid residue on an acceptor protein. Although PARP1-mediated ADP-ribosylation can occur on several amino acid residues, including aspartic acid (D), glutamic acid (E), lysine (K), serine (S), threonine (T), and tyrosine (Y), serine residues are now considered the predominant targets of PARP1-mediated ADP-ribosylation during the DNA damage response [14–16]. This understanding emerged following studies showing that histone PARylation factor 1 redirects PARP1 substrate specificity from acidic residues, particularly aspartate and glutamate, toward serine residues [14,16–19]. Following initiation, PARP1 elongates PAR chains through the sequential addition of ADP-ribose units to the terminal ADP-ribose of the growing chain, generating linear PAR polymers connected mainly by a 2′-1″ ribose–ribose glycosidic bond. PARP1-generated PAR polymers vary in length, ranging from short oligomers to long chains that can exceed 200 ADP-ribose units [13]. During elongation, PARP1 can also introduce branch points into the growing PAR chain by forming alternative ribose–ribose glycosidic linkages, primarily 2″-1″ bonds, giving rise to branched PAR structures (Figure 1B) [13]. Branched PAR structures increase the number of interaction sites available to PAR-binding proteins. Accordingly, electron microscopy and atomic force microscopy studies have shown that PARylated PARP1 can adopt a star-like conformation decorated with multibranched PAR chains [20–22].

### ADP-ribosylation readers

The recruitment of PAR-binding proteins is mediated by a wide variety of binding modules, including macrodomains, tryptophan-tryptophan-glutamate (WWE) domains, poly(ADP-ribose)-binding zinc-finger (PBZ) domains, PAR-binding motifs (PBMs), FHA/BRCT domains, and PIN domains [23,24]. These domains differ in their specificity and affinity for distinct features of PAR. For example, PBZ domains recognize linear PAR chains by binding ADP-ribose-ADP-ribose junctions, whereas macrodomains preferentially bind terminal ADP-ribose residues, and WWE domains recognize the iso-ADP-ribose moiety within PAR chains (Figure 1B) [23]. Beyond these dedicated PAR readers, the structural similarity between PAR and RNA enables PAR to interact with numerous RBPs. By mimicking the negative charge and repetitive structure of oligonucleotides, PAR can engage RNA-binding surfaces present in a subset of RBPs, such as RNA-recognition motifs (RRMs), RG/RGG repeats, serine/arginine-rich repeats, and OB-fold domains [10,25]. Indeed, accumulating evidence indicates that several RRM- or RG/RGG-containing proteins, including SRSF1, NONO, ALYREF, DDX21, HTATSF1, PABPN1, NCL, and FUS, interact with PAR chains (Table 1) [10,26–29]. Structural modeling of the PABPN1 RRM domain suggests that RNA and PAR bind the same electropositive surface and share two adenine-binding pockets [10]. Proteins containing RG/RGG repeats bind PAR chains primarily through electrostatic and hydrogen-bonding interactions between the positively charged guanidinium groups of arginine residues and the negatively charged diphosphate groups of PAR [25].

**Table 1:** RBPs with reported PAR binding

RNA-binding protein	PAR- binding domain	Evidence for PAR binding	Functional consequence	References
ALYREF	RGG	RGG-rich region of ALYREF is required for PAR-dependent recruitment. But direct binding of the RGG domain to PAR requires experimental validation	PARP-dependent recruitment to DNA damage	[30,31]
CHTOP	RGG	Direct binding of the RGG domain to PAR requires experimental validation	PARP-dependent recruitment to DNA damage	[30]
CIRBP	RGG	RGG binds PAR as detected by dot-blot assay.	PARP-dependent recruitment to DNA damage.	[32]
DDX21	CTD	CTD mutant reduces PAR binding as monitored by dot-blot assay	PARP1-dependent regulation of DDX21 nucleolar localization	[30,33]
DEK	PBD	PBD2 binds PAR as measured by slot-blot assay	RPA foci formation in response to hydroxyurea	[34]
EWS	RGG	EWS associates with PARylated PARP1 as detected by western-blot	PARP-dependent recruitment to DNA damage.	[35,36]
FBL	RGG	GAR deletion reduces PAR binding as monitored by western-blot	PARP-dependent recruitment to DNA damage	[37]
FUS	RGG	RGG2 domain binds PAR as detected by dot-blot	PARP-dependent recruitment to DNA damage	[35,38,39,40]
	RRM	Direct PAR–RRM interaction as detected by chemical shift perturbation	Increased cellular PAR levels	[26]
G3BP1	RGG	GAR mediates association of G3BP1 with PAR monitored by ^32^P-pADPr peptide-array.	Promotes stress granule formation	[41]
hnRNP A1	PBM	PBM mediates association of hnRNP1 with PARylated proteins as monitored by dot-blot.	Stress granule association	[42]
hnRNP A2	PBM	PBM binds PAR as detected by dot-blot assay.	Unknown	[43]
HuR	PBM	PBM mediates association of HuR with PAR monitored by ^32^P-pADPr peptide-array	PAR-dependent regulation of HuR localization/function	[44]
METTL14	RGG	METTL14 binds PAR as measured by EMSA	PARP-dependent recruitment to DNA damage	[45]
NONO	RRM	RRM1 domain binds PAR as detected by dot-blot.	PARP-dependent recruitment to DNA damage	[29,30]
Nucleolin	RRM	RRM2-4 binds PAR as detected by PAR pull-down.	PARP-dependent recruitment to DNA damage.	[10]
PABPN1	RRM	RRM binds PAR as detected by PAR pull-down.	PARP-dependent recruitment to DNA damage.	[10]
PARP13	WWE	WWE2 binds to ADPr as identified by X-Ray crystallography	Antiviral activity	[46]
SAF-A (hnRNPU)	RGG	RG binds PAR as detected by PAR-binding assay	PARP-dependent recruitment to DNA damage.	[47]
SFPQ	RRM	Direct binding of the RRM domain to PAR requires experimental validation	PARP-dependent recruitment to DNA damage.	[30,48]
SRSF1	RRM and RS	RRM1 and RS domains bind PAR as detected by slot-blot assay.	PAR binding inhibits TOP1-mediated SRSF1 phosphorylation	[28]
TAF15	RGG	RGG-rich region of TAF15 is required for PAR-dependent recruitment. But direct binding of the RGG domain requires experimental validation	PARP-dependent recruitment to DNA damage.	[35,49]
TDP-43	PBM	Mutation of PBMs reduces PAR binding as detected by dot blot.	Stress granule association	[50]
YB-1	CTD	CTD binds PAR as measured by EMSA	Regulates PARP1	[51]

### ADP-ribosylation erasers

ADP-ribosylation erasers terminate ADP-ribose-dependent signaling by reversing MARylation and PARylation, preventing aberrant persistence of MAR and PAR following DNA damage [52,53]. These enzymes hydrolyze specific chemical bonds either within PAR polymers or between the proximal ADP-ribose unit and its acceptor amino acid, conferring complementary substrate and linkage specificities (Figure 1B). PARG is the main enzyme responsible for degrading PAR chains synthesized by PARP1 [54]. Its catalytic macrodomain primarily functions as an exoglycosidase, sequentially removing ADP-ribose units from the protein-distal end of PAR chains [55,56]. In addition, PARG also has an endoglycosidic activity that cleaves internal ribose–ribose linkages, including branch points, to generate shorter protein-free PAR fragments (Figure 1B). However, PARG cannot hydrolyze the bond connecting the proximal ADP-ribose unit to the modified protein, leaving a residual MAR modification on the target protein [57]. Complete removal of PARP1-mediated ADP-ribosylation requires additional nuclear erasers, including ARH3, and the macrodomain-containing enzymes TARG1 and MacroD1 (Figure 1B). ARH3 hydrolyzes O-linked ADP-ribose from serine residues, and because serine is the predominant target of PARP1-mediated ADP-ribosylation during DNA damage, ARH3 serves as its principal eraser [19,58]. In contrast, MacroD1 and TARG1 hydrolyze ADP-ribose modifications on aspartate and glutamate residues [53]. MacroD1 removes MAR, whereas TARG1 can release both MAR and PAR [59,60]. Together, these complementary activities enable complete reversal of structurally diverse ADP-ribose modifications.

## Mechanisms of PARP1 hyperactivation

Many types of DNA damage recruit and activate PARP1. However, the extent of PARP1 activation varies greatly depending on the nature of the DNA lesion. DNA-damaging agents that predominantly induce base lesions repaired by the base excision repair (BER) pathway, including the alkylating agents methyl methanesulfonate (MMS) and N-methyl-N′-nitro-N-nitrosoguanidine (MNNG), as well as oxidative stress induced by hydrogen peroxide (H_2_O_2_), trigger PARP1 hyperactivation and high levels of PARylation [61–63]. More recently, ATR inhibitors were also shown to induce PARP1 hyperactivation by promoting BER intermediates at replication forks [64]. In contrast, DNA-damaging agents that primarily induce DNA lesions repaired through other DNA repair pathways, including ionizing radiation, topoisomerase I and II inhibitors (camptothecin [CPT] and etoposide), hydroxyurea (HU), and mitomycin C, induce low levels of PARylation [64]. All these treatments induce extensive DNA damage, indicating that the overall level of damage does not necessarily correlate with PARP1 activation and that PARP1 hyperactivation is instead triggered by specific types of DNA lesions.

The response to ATR inhibition provides a clear mechanistic example of how lesion identity dictates PARP1 hyperactivation. Acute ATR inhibition triggers a rapid surge in origin firing during S phase, increasing the number of active ongoing replication forks (Figure 2A) [65–67]. Because replication forks require replication protein A (RPA) to protect exposed single-stranded DNA (ssDNA) on the lagging strand, excessive origin firing rapidly exhausts the available pool of nuclear RPA, leaving unprotected ssDNA [66]. Unprotected ssDNA becomes vulnerable to deamination by the cytosine deaminases APOBEC3A (A3A) and APOBEC3B (A3B), which convert cytosines into uracils [64,68,69]. These uracils are then removed by the uracil DNA glycosylase UNG2, generating abasic sites, also known as apurinic/apyrimidinic (AP) sites [64,68,70]. AP sites are subsequently cleaved by apurinic/apyrimidinic endonuclease 1 (APE1), causing replication fork collapse, DSB formation, and PARP1 hyperactivation (Figure 2A) [64,71,72]. Biochemically, APE1-mediated cleavage of AP sites generates DNA breaks bearing 5′-deoxyribose phosphate (5′-dRP) ends (Figure 2A) [73,74]. Because 5′-dRP ends are high-affinity substrates for PARP1 binding and activation [75–77], APE1-generated 5′-dRP intermediates have been proposed to drive PARP1 hyperactivation following ATRi [64]. This model also explains why other forms of replication stress fail to induce PARP1 hyperactivation despite generating extensive DNA damage. For example, CPT-mediated inhibition of topoisomerase I generates SSBs that are converted directly into DSBs when encountered by active replication forks [78–80], whereas HU depletes deoxyribonucleotide pools, causing replication fork stalling and MUS81-dependent fork cleavage [81–83]. Although both treatments generate replication-associated DSBs, neither generates APE1-dependent 5′-dRP intermediates, resulting in substantially lower PARP1 activation [64].

**Figure 2:**
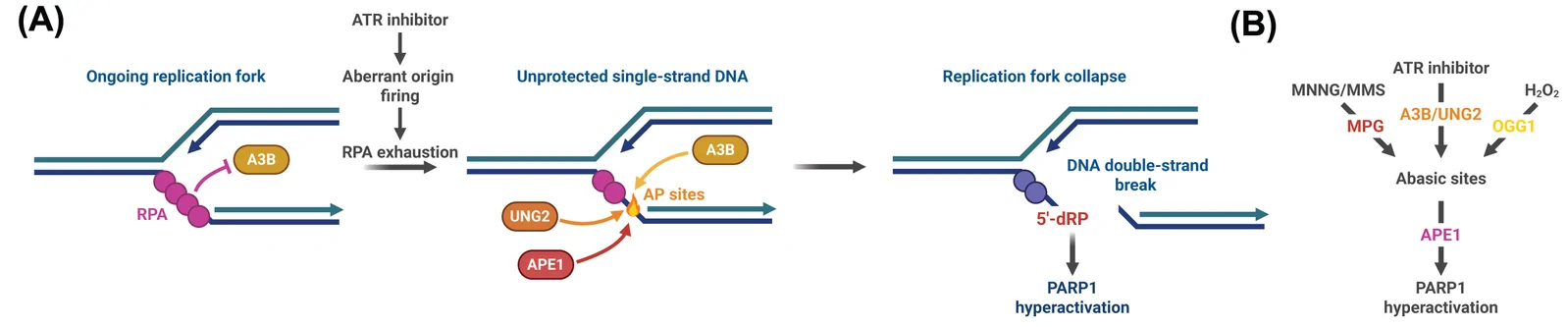
Mechanisms of PARP1 hyperactivation (**A**) During ongoing DNA replication, RPA coats and protects ssDNA at replication forks from A3B-mediated deamination. However, ATR inhibition triggers excessive origin firing, depleting the available RPA pool and exposing ssDNA to A3B activity. The resulting uracils are excised by UNG2, generating abasic sites that APE1 cleaves to produce 5′-dRP intermediates, which drive PARP1 hyperactivation. (**B**) MNNG, MMS, ATR inhibition, and H_2_O_2_ induce PARP1 hyperactivation through distinct mechanisms that converge on abasic site formation. Although the initiating DNA lesions are processed by different DNA glycosylases, the resulting AP sites are cleaved by APE1, leading to PARP1 hyperactivation.

The same biochemical concept extends to other DNA-damaging agents. PARP1 hyperactivation induced by MMS, MNNG, or H_2_O_2_ also depends on APE1 activity (Figure 2B), despite being independent of A3A/B and UNG2 [64]. Instead, MPG excises alkylated bases generated by MMS and MNNG, whereas OGG1 removes oxidative lesions induced by H_2_O_2_ [64]. Both pathways generate AP sites that are subsequently cleaved by APE1, producing SSBs with 5′-dRP termini [64]. Thus, regardless of the pathway by which AP sites are generated in response to different forms of genotoxic stress, their cleavage by APE1 and the resulting formation of 5′-dRP ends appear to be the critical steps that drive PARP1 hyperactivation (Figure 2B).

5′-dRP is a toxic repair intermediate, or “dirty end,” that must be removed before DNA synthesis and ligation occur to complete BER [84]. During short-patch BER, DNA polymerase β (Polβ) removes the 5′-dRP group through its dRP lyase activity and then fills the single-nucleotide gap [85]. Alternatively, when the 5′-dRP group cannot be efficiently removed by the dRP lyase activity of polβ, repair can proceed through long-patch BER, in which strand-displacement synthesis generates a 5′ flap that is cleaved by flap endonuclease 1 [86]. PARP1 promotes the resolution of these BER intermediates by binding to SSBs and recruiting repair factors to the site of damage [87]. Upon activation, PARP1 autoPARylation facilitates the recruitment of the scaffold protein XRCC1 and the chromatin remodeler ALC1. In turn, XRCC1 coordinates the assembly of several BER factors involved in SSB repair, including polβ [88], DNA ligase III (LIG3) [89], aprataxin [90], and polynucleotide kinase 3′-phosphatase [91]. In parallel, ALC1 remodels the surrounding chromatin to increase DNA accessibility and facilitate the recruitment and activity of the DNA repair machinery [92]. Therefore, the strong affinity of PARP1 for 5′-dRP ends may reflect the need to efficiently recruit polβ and other repair factors to rapidly remove these toxic intermediates from DNA and complete BER. Importantly, persistent engagement of PARP1 at these intermediates may itself contribute to PARP1 hyperactivation. Consistent with this idea, XRCC1 and Polβ promote PARP1 dissociation from DNA lesions, preventing toxic PARP1 trapping and hyperactivation [64,93–95]. XRCC1- and Polβ-mediated release of PARP1 is essential for downstream BER factors to access DNA breaks and complete repair [93,95].

## Cellular consequences of PARP1 hyperactivation

### Regulation of biomolecular condensates by PAR

#### PAR as a dynamic regulator of biomolecular condensates

PAR is a highly negatively charged, multivalent polymer that functions as a dynamic scaffold for biomolecular condensation (Figure 3) [11,96]. These biomolecular condensates assemble through networks of weak, dynamic, and multivalent interactions, where individual molecules contain multiple binding sites that can engage several partners simultaneously, including proteins and biopolymers such as DNA, RNA, and PAR [96]. Collectively, these interactions drive liquid–liquid phase separation (LLPS), a process that dynamically concentrates soluble molecules into spatially defined, membraneless compartments [97].

**Figure 3:**
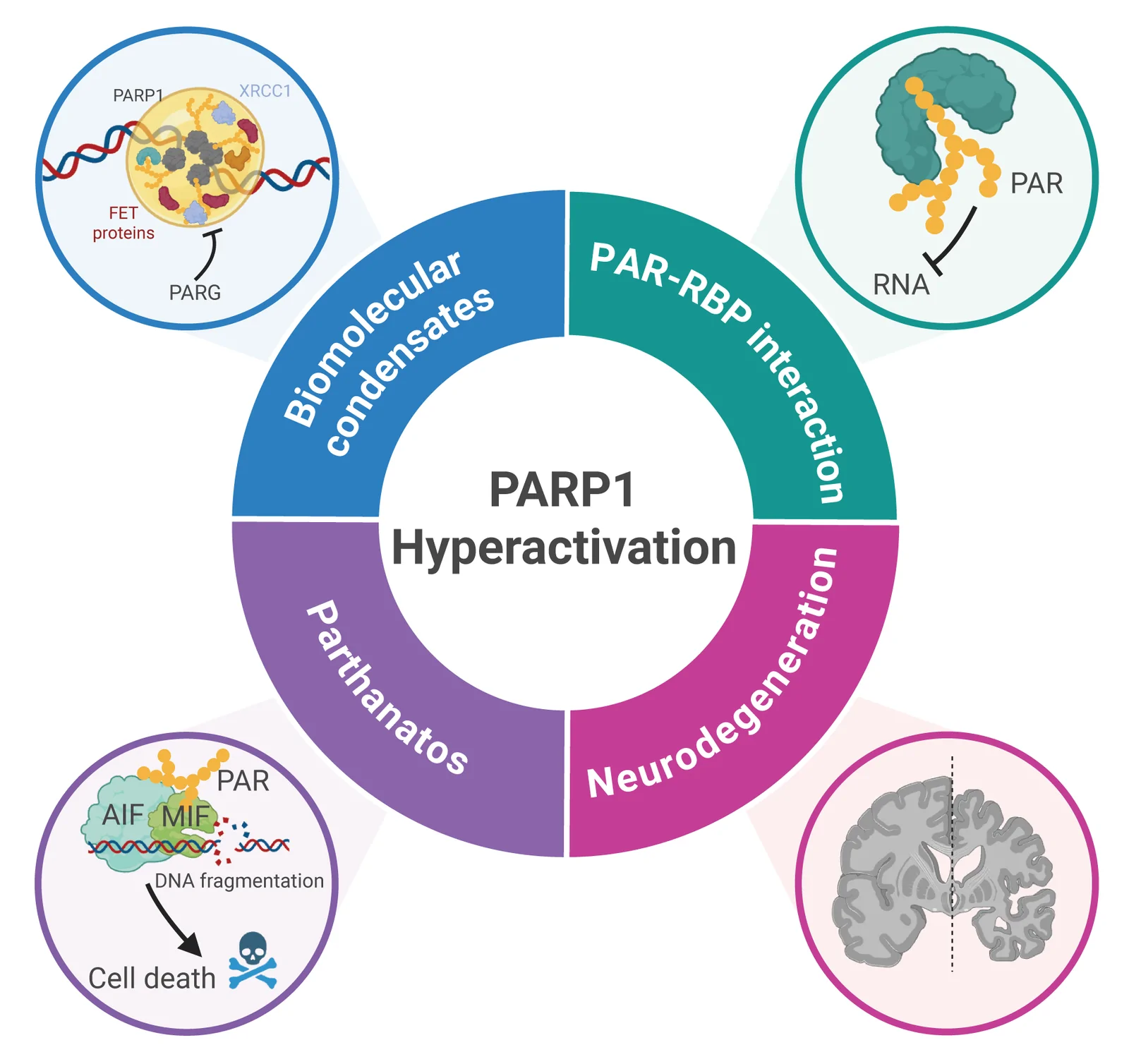
PARP1 hyperactivation drives multiple PAR-dependent cellular responses PAR promotes the assembly of biomolecular condensates, competes with RNA for binding to specific RBPs, and triggers parthanatos through AIF/MIF-dependent genome fragmentation. Persistent PARP1 hyperactivation also contributes to several neurodegenerative diseases.

Because PAR shares key physicochemical properties with nucleic acids, RNA serves as an excellent model for understanding how these polymers drive condensation. RNA is a major structural and regulatory component of many biomolecular condensates due to its polymeric architecture, negative charge, and inherent multivalency [98]. RNA molecules can form RNA–RNA interactions, adopt complex secondary and tertiary structures, and bind multiple RBPs simultaneously [98]. RBPs further contribute to this multivalency through intrinsically disordered regions (IDRs), including low-complexity domains (LCDs), which are repetitive, often charged sequences that mediate protein–protein and protein–RNA interactions, and through oligomerization domains that assemble multiple protein subunits around RNA [99]. These interactions generate highly interconnected molecular networks that promote condensate formation. Like RNA, a PAR chain with repeating ADP-ribose units creates numerous binding sites, allowing a single PAR chain to engage multiple PAR-binding proteins simultaneously [11,96]. However, unlike RNA, which is constitutively present in cells, PAR is synthesized rapidly and transiently following PARP1 activation. This allows cells to precisely and dynamically regulate the spatial and temporal assembly of biomolecular condensates in response to DNA damage.

#### PARP1 and PARG control condensate assembly and disassembly

Early evidence that PAR can promote biomolecular condensation emerged from studies using UV-laser microirradiation to induce localized DNA damage, especially base lesions and SSB, which are repaired by BER and cause PARP1 hyperactivation [35,38,100,101]. The resulting accumulation of PAR at damage sites seeds the condensation by LLPS of FET family proteins (FUS, EWS, and TAF15), which contain RGG-rich domains [35,101,38]. FET-containing condensates form at DNA lesions within minutes of microirradiation but are short-lived, dissipating as PARG degrades PAR chains [102]. Moreover, PARG inhibition promotes PAR-dependent formation of FET condensates at replication forks, causing fork slowing and reversal [103]. This PARP1-dependent response extends beyond FET proteins, as many other RBPs, including NONO, SFPQ, ALYREF, THRAP3, DDX21, HNRNPU, and CHTOP, also accumulate at microirradiation-induced lesions (Table 1) [29,49,30]. These proteins contain PAR-binding domains, including RGG repeats and RRMs, together with IDRs, that enable multivalent interactions with PAR chains and other factors, promoting condensation via LLPS. Importantly, PAR recognition is not a general property of RBPs, and biochemical studies indicate that only a subset of RNA-binding domains recognize PAR [10]. Many of these PAR-recruited RBPs have established roles in DNA damage signaling and repair [104], indicating that PAR-dependent RBP recruitment is important for the DNA damage response (Table 1). Nevertheless, because UV-laser microirradiation generates supraphysiological levels of localized DNA damage and PAR synthesis, it remains unclear whether all RBPs recruited under these conditions make physiologically relevant contributions to genome maintenance. Despite this limitation, laser microirradiation remains a powerful experimental system for dissecting the molecular principles governing PAR-dependent condensate assembly.

Recent studies have shown that not only PAR but also PARP1 itself promotes the formation of biomolecular condensates through DNA-dependent co-condensation [105,106]. *In vitro* reconstitution experiments demonstrated that DNA end recognition by the PARP1 zinc-finger domains promotes condensate formation. These condensates generate bidirectional mechanical forces that help maintain DNA ends in close proximity, potentially helping cells to keep DSBs together [105]. Catalytic activation of PARP1 subsequently remodels these condensates through PARylation, which can either stabilize or destabilize the assembly depending on the extent of PARylation [105,106]. Moderate PARylation promotes the recruitment of FET family members, which stabilize PARP1–DNA condensates and counteract PARylation-mediated dissolution. In contrast, extensive PARP1 autoPARylation weakens PARP1–DNA interactions, leading to condensate dissolution and the release of PARP1 from DNA [105,106]. The effect of PARG depends on the level of PAR: following extensive PARylation, PARG can restore PARP1–DNA condensation [105], whereas upon low PARylation, PARG-mediated PAR degradation suppresses condensate formation [106]. It was proposed that extensive PARylation caused a highly negatively charged environment that weakens the multivalent interactions required for condensate assembly, although this mechanism remains to be established.

#### Excessive PAR signaling promotes persistent and non-productive condensates

In cells, prolonged PARG inhibition induces the formation of PARP1- and PAR-dependent nuclear condensates enriched in BER factors, including XRCC1, LIG3, and POLβ [107]. Although these condensates are not associated with persistent DNA damage lesions, their formation depends on UNG2 [107]. This suggests that BER processing of these uracil lesions could generate similar APE1-dependent 5′-dRP-containing intermediates, leading to PARP1 hyperactivation (see the ‘Mechanisms of PARP1 hyperactivation’ section). Microirradiation further enhanced the formation of nuclear PARP1 condensates in PARG-inhibited cells [108,109]. Under these conditions, hyper-PARylated PARP1 accumulates in nuclear bodies spatially separated from the sites of DNA damage generated by microirradiation [108,109]. Because PAR is not efficiently degraded, these condensates persist even after the initiating lesions have been resolved, sequestering repair factors that would otherwise remain available for recruitment to other sites of DNA damage. Thus, PARG promotes condensate disassembly by degrading PAR, terminating multivalent PAR-mediated interactions, and returning PARP1 and its associated factors to the soluble nuclear pool. Conversely, PARG inhibition prolongs their sequestration in non-productive condensates, impairing repair-factor recruitment at DNA lesions, and potentially reducing DNA repair efficiency.

PARylation also controls the selective recruitment and spatial organization of DNA repair factors within PARP1 condensates. POLβ and FUS partition relatively homogeneously within PARP1 condensates [105,106]. In contrast, XRCC1 and LIG3 display heterogeneous spatial distributions, forming distinct substructures within multiphase PARP1 condensates [105,106]. These findings suggest that PARP1 condensation provides an initial DNA-dependent scaffold, whereas PAR synthesis dynamically remodels condensate material properties and promotes the selective and spatially organized recruitment of DNA repair factors [105,106]. Moreover, low concentrations of PAR promote FUS condensation far more efficiently than RNA, which requires substantially higher concentrations to induce FUS condensation [110]. In contrast with RNA, which remains associated with FUS condensates and contributes to their structural scaffold, PAR interacts only transiently with the RGG domains of FUS [110], which is thought to favor intermolecular FUS–FUS interactions between its LCD, triggering FUS oligomerization and nucleating condensate formation, without PAR chains serving as a scaffold for the condensates. Therefore, PAR can promote condensate formation through two non-mutually exclusive mechanisms: by acting as a multivalent scaffold that locally concentrates PAR-binding proteins and supports cooperative interactions, or by destabilizing their soluble states and thereby lowering the nucleation barrier for phase separation. The relative contribution of these mechanisms is likely determined by PAR abundance, chain length, and branching, as well as the concentration and interaction properties of the recruited proteins.

### PARP1 hyperactivation rewires nuclear RBP–RNA interactions

PARP1 hyperactivation profoundly alters the interactions between RBPs and their RNA substrates, leading to a reorganization of nuclear RBP–RNA interactions (Figure 3). For example, acute MNNG treatment causes ALYREF to dissociate from RNA, and this effect is restored by co-treatment with a PARP inhibitor, demonstrating that disruption of ALYREF–RNA interaction depends on PARP1 activity [30]. Both the affinity of these RBPs for PAR and their PARylation are likely to contribute to this process. First, ALYREF is recruited to laser-induced DNA lesions in a PARP1-dependent manner through its RGG-rich RNA-binding domains, indicating that PAR can compete with RNA for RBP binding [30, 31]. Similar PARP1-dependent recruitment of CHTOP and DDX21 was observed upon microirradiation. Second, ALYREF, CHTOP, and DDX21 were identified by mass spectrometry as major PARP1-PARylated targets following genotoxic stress [9,111], raising the possibility that their PARylation further enhances PAR chains’ ability to compete with RNA by positioning highly negatively charged polymers near RNA-binding domains. Together, these complementary mechanisms could explain how PARP1 hyperactivation reorganizes nuclear RBP function while producing distinct effects on individual proteins, depending on their relative capacity to bind PAR and undergo PARylation.

An important consequence of PARP1-mediated remodeling of RBP–RNA interactions is defective nuclear RNA splicing and mRNA export [30]. PAR binding to multiple RBPs involved in RNA splicing results in widespread exon and intron retention following ATR inhibition or MNNG treatment in a PARP1-dependent manner. Moreover, consistent with the disruption of the TREX mRNA export factors ALYREF and CHTOP [112,113], PARP1 hyperactivation impairs RNA export from the nucleus to the cytoplasm, leading to nuclear RNA retention (Figure 4). As a result, the pool of RNA available for translation in the cytoplasm decreases, leading to translation arrest [30]. At the same time, the retained nuclear RNAs aggregate in PARP1-mediated nuclear condensates, which are enriched in mRNA and nuclear speckle factors [30]. One possible explanation is that RNAs no longer bound by their associated RBPs engage in aberrant multivalent interactions with other nuclear RBPs and nuclear speckle-associated factors, promoting condensate assembly and growth. This model is similar to cytoplasmic stress granule assembly. In response to cellular stresses that activate the integrated stress response, suppression of translation initiation causes non-translating, ribosome-free mRNAs to accumulate in the cytoplasm [114,115]. These untranslated naked mRNAs are bound by RBPs such as G3BP1/2, TIA-1, and other low-complexity RBPs, whose multivalent interactions drive LLPS and stress granule formation [114,115]. Similarly, PARP1 hyperactivation promotes the accumulation of improperly assembled nuclear ribonucleoproteins, exposing RNA- and protein-binding surfaces that can engage in aberrant multivalent interactions with nuclear speckle components and other RBPs, promoting nuclear condensate formation. Notably, PAR is not enriched in nuclear condensates, suggesting that it acts as a transient nucleator rather than a structural component of the mature condensate [30]. By destabilizing native RBP–RNA interactions, PAR generates condensation-prone RNAs and RBPs, whereas retained RNAs provide a multivalent scaffold that enhances condensate formation (Figure 4). The absence of PAR from these nuclear condensates may also reflect the relatively short PAR chains produced following ATRi or MNNG treatment [64], whose length and lifetime are constrained by PARG activity. Thus, PARP1-driven condensate formation may represent a sequential mechanism in which transient PAR signaling initiates RBP remodeling, whereas RNA subsequently acts as the principal scaffold for condensate assembly.

**Figure 4:**
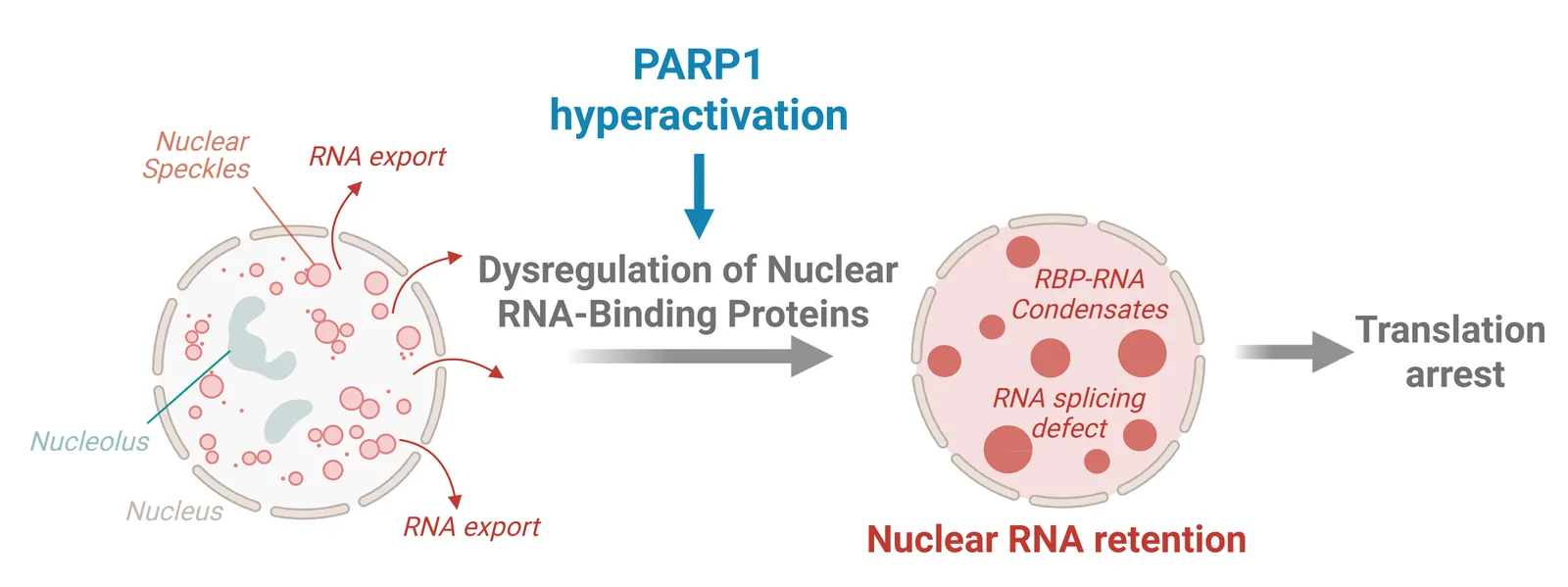
PARP1 hyperactivation causes nuclear RNA retention, leading to translation arrest High levels of PARylation destabilize RBPs involved in RNA splicing and export by disrupting their association with RNA. This global RBP reorganization causes splicing defects and nuclear RNA retention, reducing mRNA export to the cytoplasm and limiting the pool of transcripts available for translation.

### Parthanatos

Although transient PARP1 activation is essential for DNA repair, sustained PARP1 hyperactivation becomes cytotoxic and can trigger a regulated form of cell death known as parthanatos (Figure 3). This pathway is induced by DNA-damaging agents that strongly activate BER, including MNNG, MMS, and H_2_O_2_ [116,117]. Unlike canonical apoptosis, parthanatos proceeds independently of caspase activation and typically does not involve the formation of an apoptotic body. Although PARP1 hyperactivation consumes large amounts of NAD^+^ and can consequently impair ATP production, metabolic depletion alone does not fully account for parthanatos [116,117]. Instead, excessive accumulation of PAR acts as a central pro-death signal. Following PARP1 hyperactivation, PAR polymers produced in the nucleus translocate to the mitochondria, where they bind apoptosis-inducing factor (AIF) and promote its release into the cytosol [118,119]. After its release, AIF associates with macrophage migration inhibitory factor (MIF), also known in this context as the parthanatos-associated AIF nuclease [120,121]. The AIF–MIF complex subsequently enters the nucleus, where MIF uses its nuclease activity to induce extensive, large-scale genomic DNA fragmentation, resulting in irreversible cell death [120]. Genetic deletion or inhibition of MIF nuclease activity suppresses parthanatos, supporting its role as a terminal executioner of this pathway [120,122]. In turn, parthanatos promotes the release of damage-associated signals that activate microglia and astrocytes, thereby amplifying neuroinflammation [123–127]. Thus, parthanatos has been implicated in several neurodegenerative diseases, including Alzheimer's disease, Parkinson's disease, Huntington's disease, and amyotrophic lateral sclerosis [116,122,128].

### Genetic disease associated with PARP1 hyperactivation

Pathological PARP1 hyperactivation is also increasingly recognized as a driver of human disease, particularly neurodegeneration (Figure 3). Genetic disorders that either enhance PARP1 activation or impair PAR turnover provide compelling evidence that chronic PAR signaling is intrinsically toxic. For example, biallelic XRCC1 mutations impair SSB repair, resulting in PARP1 hyperactivation and elevated PAR levels that cause neurological dysfunction, including oculomotor apraxia and progressive cerebellar ataxia [94,129]. Similarly, XRCC1-deficient mice exhibit dysregulated neuronal signaling, lethal seizures, and a shortened lifespan, all of which can be prevented by treatment with PARP inhibitors [130]. Persistent PARP1 hyperactivation has also been reported in Nijmegen breakage syndrome, a rare inherited disorder caused by hypomorphic mutations in NBN, a component of the MRE11-RAD50-NBN DNA repair complex [131]. NBN-deficient cells exhibit hyperactivation of PARP1 and NAD+ depletion, leading to increased reactive oxygen species [131]. Defects in PAR turnover similarly result in neurological disease. Biallelic mutations in *ADPRS*, which encodes ARH3, are associated with severe neurodegeneration in humans and with the accumulation of MAR species in cells [132–134]. Patients with ARH3 mutations develop a neurodegenerative disorder that typically manifests during childhood or adolescence and is characterized by progressive brain atrophy, developmental delay or regression, seizures, ataxia, and axonal sensorimotor neuropathy [132–134]. Likewise, mutations in TARG1 are also associated with severe neurodegeneration, resulting from the impaired removal of PARP1-mediated mono-ADP-ribosylation from glutamate and aspartate residues [135]. Consistent with the importance of PAR turnover, loss of PARG in *Drosophila* melanogaster causes progressive neurodegeneration, reduced locomotor activity, and a shortened lifespan [136], phenotypes similar to those associated with XRCC1 mutations in humans. Together, these findings indicate that tight control of PARP1 activity is essential for limiting the neurotoxic consequences of aberrant PARP1 activity and that suppressing pathological PARP1 hyperactivation can prevent or alleviate the associated neurological phenotypes. However, demonstrating that genetic or pharmacological suppression of PARP1 rescues neurodegeneration across disease-relevant models or that PARP inhibition provides clinical benefit in affected patients will be necessary to establish PARP1 hyperactivation as a causal and therapeutically actionable driver of these disorders.

## Conclusion

PARP1 hyperactivation illustrates how a normally protective stress response can become pathogenic when its activity is no longer properly controlled. Whereas transient PARP1 activity promotes DNA repair, sustained PARP1 hyperactivation disrupts cellular homeostasis and induces cell death. The magnitude of PARP1 activation is determined not by the amount of DNA damage but by a specific type of repair intermediate mediated by APE1-dependent cleavage of abasic sites. Excessive PAR synthesis has broad consequences for nuclear organization, disrupting RNA biogenesis, including RNA export and splicing, promoting aberrant condensate formation, and triggering cell death through parthanatos. Consistent with this model, mutations that prolong PARP1 activity at DNA lesions or impair ADP-ribose turnover lead to pathological PARP1 hyperactivation and neurodegeneration.

## Perspectives

- PARP1 is a key DNA repair factor, yet its hyperactivation converts a genome-protective response into pathological signaling that rewires RNA biogenesis and triggers cell death, illustrating its dual role in genome maintenance and disease.
- Contrary to the common view that PARP1 activation scales with the overall level of DNA damage, different lesion types activate PARP1 to different extents. APE1-mediated cleavage of abasic sites generates DNA breaks with 5′-dRP ends, representing the critical convergent step driving PARP1 hyperactivation under genotoxic stress.
- PARP1 hyperactivation profoundly disrupts RNA biogenesis, including RNA export and splicing, promotes aberrant biomolecular condensate formation, and triggers parthanatos. However, the molecular mechanisms linking aberrant PARP1 activity to these cellular outcomes remain not fully understood.

## Open Access

Open access for this article was enabled through an open access agreement between the Biochemical Society/Portland Press and the University of California.

## Abbreviations

A3A: APOBEC3A
A3B: APOBEC3B
AIF: apoptosis-inducing factor
AP: apurinic/apyrimidinic
APE1: apurinic/apyrimidinic endonuclease 1
BER: base excision repair
CPT: camptothecin
DSBs: double-strand breaks
5′-dRP: 5′-deoxyribose phosphate
H_2_O_2_: hydrogen peroxide
HU: hydroxyurea
IDRs: intrinsically disordered regions
LIG3: ligase III
LLPS: liquid–liquid phase separation
LCDs: low-complexity domains
MMS: methyl methanesulfonate
MIF: migration inhibitory factor
MNNG: N-methyl-N′-nitro-N-nitrosoguanidine
PAR: poly(ADP-ribose)
PBMs: poly(ADP-ribose)-binding motifs
PBZ: poly(ADP-ribose)-binding zinc-finger
PARG: poly(ADP-ribose) glycohydrolase
PARP1: poly(ADP-ribose) polymerase 1
Polβ: polymerase β
RBPs: RNA-binding proteins
RRMs: RNA-recognition motifs
RPA: replication protein A
SSBs: single-strand breaks
ssDNA: single-stranded DNA
WWE: tryptophan-tryptophan-glutamate
